# Improved survival over time with immunotherapy in locally advanced and metastatic cutaneous squamous cell carcinomas

**DOI:** 10.1007/s00432-023-05593-2

**Published:** 2024-03-16

**Authors:** Sophie Schneider, Thomas Ferte, Océane Ducharme, Léa Dousset, Sorilla Prey, Caroline Dutriaux, Emilie Gerard, Marie Beylot-Barry, Anne Pham-Ledard

**Affiliations:** 1 Dermatology Department, Hôpital Saint André, CHU Bordeaux, 1 Avenue Jean-Burguet, 33000 Bordeaux, France; 2 Public Health Centre, Methodological Support Unit for Clinical and Epidemiological Research, CHU Bordeaux, 33000 Bordeaux, France; 3 INSERM U1312, BRIC, Team 5 Translational Research on Oncodermatology and Rare Skin Diseases, University Bordeaux, 33076 Bordeaux, France

**Keywords:** Advanced cutaneous squamous cell carcinoma, Immunotherapy, Chemotherapy, Check-point inhibitor, Epidemiology, Anti-programmed-death 1

## Abstract

**Purpose:**

Cutaneous squamous cell carcinoma (cSCC) is the second most common cancer in white-skinned populations. There is little information on the epidemiology of cSCC, and even less on advanced cases (acSCC). Therefore, we analyzed acSCC patients to describe their characteristics, management, and outcomes over time.

**Methods:**

A single-center retrospective study was conducted over a period of 5 years, including all patients who started systemic therapy for acSCC. The patient characteristics, cSCC management, response to therapy, and survival were recorded. Patients were stratified into equal chronological periods (periods 1 and 2). A subgroup analysis was performed to compare patients who received immunotherapy (group 1) with those who did not (group 2).

**Results:**

The study included 127 patients, and patient numbers increased by an average of 19.7% per year. Most patients were male (88/127), elderly (mean 81.6 years), with comorbidities, and 27.6% were immunocompromised. The median overall survival (OS) was higher in period 2 (20 months) than in period 1 (10 months) (hazard ratio [95% confidence interval] = 0.62 [0.39; 0.98], *p* = 0.04). The risk of progression increased with age and immunosuppression. Of the 64 patients who received second-line therapy, 38 had immunotherapy (group 1) and 26 received other therapies (group 2). Immunotherapy reduced mortality and progression by 71% (*p* = 0.004) and 67% (*p* = 0.002), respectively.

**Conclusions:**

Patients with acSCC are usually very frail and elderly. OS increased over time, with a twofold improvement between periods 1 and 2, whereas progression-free survival (PFS) did not increase. Access to immunotherapy reduced mortality in a majority of patients in period 2. Immunosuppression and advanced age were associated with lower PFS.

## Introduction

Skin carcinomas are the most common human cancers. Cutaneous squamous cell carcinoma (cSCC) is the second most common type after basal cell carcinoma, and its dissemination capacity causes significant morbidity, resulting in the majority of non-melanoma skin cancer (NMSC) deaths (Que et al. 2018). Solar ultraviolet radiation is the leading cause of cSCC (Boukamp 2005), and immunosuppression is the second major risk factor (Alam and Ratner 2001). cSCC incidence is increasing among white-skinned populations worldwide (Lomas et al. 2012). A study conducted by the Mayo Clinic showed a 263% overall increase in cSCC incidence from 1976–1984 to 2000–2010 (Muzic et al. 2017). Predictions suggest that cSCC incidence is likely to continue to increase (Goon et al. 2017). Despite its frequency, cSCC is usually excluded from general cancer studies and registries. In France, there are two departmental skin carcinoma registries: the Doubs (2017) and Haut-Rhin (Buemi et al. 2017) registers. Both show increased cSCC incidence, standardized on the world population, with a male predominance. Increases in cSCC incidence and mortality represent a significant financial burden; therefore, the diagnostic and therapeutic management of cSCC are a major public health issue (Vallejo-Torres et al. 2014).

Although surgery is curative in more than 90% of cases (Brougham et al. 2012), delayed or inadequate management especially in immunocompromised patients may lead to advanced stages (acSCC), locally advanced cSCC or metastatic cSCC, which require radiotherapy and/or systemic therapies (Stratigos et al. 2020). Prior to the immunotherapy era, chemotherapy and epidermal growth factor receptor (EGFR)-targeted therapies were mainly used (Maubec 2020). Since 2018, anti-programmed cell-death protein-1 (PD-1) monoclonal antibodies have emerged for the management of solid tumors, including acSCC. Cemiplimab was the first immunotherapy approved by the Food and Drug Administration (FDA) and the European Medicines Agency (EMA), followed by pembrolizumab (Zelin et al. 2021). In France, cemiplimab was used in an early access program from August 2018 to January 2021.

Due to the lack of epidemiological and demographic data and the public health impact of acSCC, we studied patients starting systemic therapy for acSCC in our referral center during a 5-year period to assess patient characteristics, acSCC details, initial management, systemic therapies, and outcomes.

## Materials and methods

### Eligibility criteria

This was a single-center retrospective study conducted at Bordeaux University Hospital over a period of 5 years. All patients with acSCC who started their first systemic therapy in the dermatology department from July 1, 2015 to July 1, 2020 were included, including those who started therapy in a clinical trial. Systemic therapy was defined as one of the following: platinum agent (carboplatin or cisplatin), 5-fluorouracil, cetuximab, panitumumab, nab-paclitaxel, paclitaxel, or PD-1 inhibitor (cemiplimab, nivolumab, or pembrolizumab). Patients were identified from the pharmacy database.

### Data management

Collected data were anonymized and protected during the study. All patients were informed about the possibility of secondary use of their data for scientific purposes and were allowed to express their opposition to this data use in accordance with French law. Data were obtained according to the Declaration of Helsinki and French law for retrospective, non-interventional, research studies and approved by the Research Ethics Committee of Bordeaux (CER-BDX-2023-56). The following patient characteristics were collected from electronic files: initial cSCC locations, histological reports, particularly R1 excision and neurotropism, gender, age, previous occupation, place of residence, comorbidities, immunosuppression, and other neoplasms. Comorbidities were used to calculate the corrected Charlson comorbidity index score retrospectively (Charlson et al. 1987; Extermann 2000). The patient’s initial management, type and duration of therapy, response to therapy, adverse events (AEs), status at last follow-up, death, and cause of death were recorded. To assess safety, AEs and the need for treatment interruption or hospitalization due to side effects were recorded. After the start of systemic therapy, other systemic treatments and reasons for modification were recorded, as well as other treatment modalities, such as surgery, radiotherapy, or local treatments.

### Statistical analyses

Quantitative variables are described as the median and interquartile range (IQR) or mean and range. Qualitative variables are described as numbers and percentages. The relationship between year and number of cSCC patients starting a first systemic therapy was analyzed using the incidence rate ratio estimated from a Poisson model; the same analysis was performed stratified by cancer localization, particularly the head and neck compared to other locations. Overdispersion was assessed using the approach proposed by Cameron and Trivedi (1990). Associations among year, first-line treatment, and department of residence were assessed using a multinomial logistic regression model. Associations among year, sex, immunosuppression, history of neoplasia, geriatric evaluation, radiotherapy, and multidisciplinary group meeting evaluation were assessed using logistic regression. Linear regression was used to evaluate associations among year, age at the beginning of treatment, and the Charlson score.

Duration of patient follow-up was defined as the time from the start of treatment to the date of the last follow-up. Progression-free survival (PFS) was defined as the time from the start of therapy to clinical progression objectivized by physician or radiological progression according to the Response Evaluation Criteria in Solid Tumors (RECIST) v1.1 (Eisenhauer et al. 2009) or death from any cause. Overall survival (OS) was defined as the time from the start of therapy for acSCC until death from any cause.

For survival analyses, we compared two periods within the 5-year study period: patients were stratified into egal chronological period 1 (July 1, 2015 to December 31, 2017) and period 2 (January 1, 2018 to July 2020). A univariate Cox model and Kaplan–Meier estimator were used to estimate the associations among PFS, OS, and the year of first systemic therapy initiation. A multivariate Cox model was used to estimate the associations among PFS, OS, and immunosuppression, delay of treatment, immunotherapy, radiotherapy, Charlson score, sex, and age. Survival curves were represented graphically using the conditional method (Kassambara et al. 2021). The proportional hazard assumption was assessed using the approach proposed by Grambsch and Therneau (1994). A subgroup analysis was performed to assess the effect of immunotherapy as a second-line treatment compared to other second-line treatments. Two groups of patients were included from the cohort: group 1 (*n* = 38) included patients with conventional chemotherapy or EGFR inhibitors (EGFRi) as first-line therapy and immunotherapy (anti-PD-1) as second-line; group 2 (*n* = 26) included patients with conventional chemotherapy or EGFRi as both first- and second-line treatment, without anti-PD-1. To limit indication bias, the effect of immunotherapy on PFS and OS was assessed using an adjusted multivariate Cox model on age at second-line treatment, sex, reason for treatment change, immunosuppression, and associated radiotherapy. In addition, a sensitivity analysis was performed by excluding transplant patients in whom immunotherapy was contraindicated at the time of the study.

## Results

### Patient numbers

**Fig. 1:**
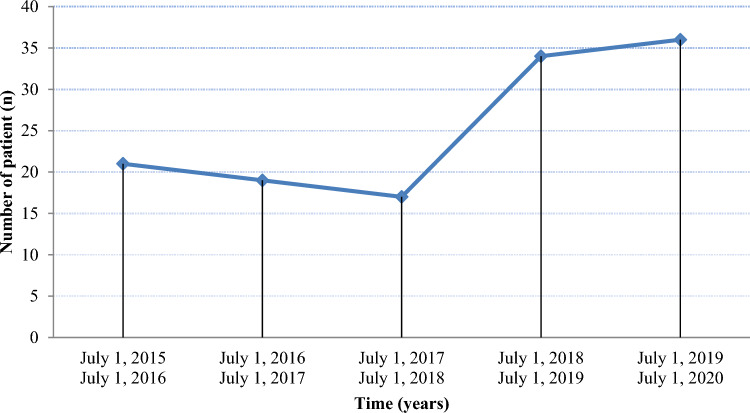
Change in the number of patients started on systemic therapy for cutaneous squamous cell carcinoma (cSCC) each year during the 5-year study period

Between July 1, 2015 and July 1, 2020, 127 patients started systemic therapy for advanced cSCC. During this period, the incidence rate ratio was 1.197 per year (95% confidence interval [CI] = [1.057; 1.359], *p* = 0.005), resulting in a mean increase of 19.7% each year, mainly concentrated in 2018 (Fig. 1). Of these 127 patients, 22 had already been included in a previous study of cemiplimab in our department (Valentin et al. 2021) and 34 in a national cohort (Hober et al. 2021).

### Patients and primary cSCC characteristics

Table 1 summarizes the patient characteristics. The mean age at diagnosis was 79.3 (range 36–95) years, including 88 men (69.3%). Their places of residence were in nine different French administrative regions, with no significant difference over the 5-year period (*p* = 0.60). Forty-one patients had a history of an occupation involving sun exposure (52.6%). Fifty-three patients had another cancer (41.7%) and 35 were immunocompromised (27.6%). The patient characteristics were comparable over the 5-year period, with no differences in gender (*p* = 0.77), age (*p* = 0.19), or immunosuppression status (*p* = 0.82). The corrected comorbidity median Charlson score was 3 (IQR, [2–4]) with a minimum score of 0 and a maximum of 14, without variation over time.

**Table 1:** Patients and primary cSCC characteristics

	2015–2016 (21)	2016–2017 (19)	2017–2018 (17)	2018–2019 (34)	2019–2020 (36)	Total (127)
*cSCC initial diagnosis age, years*						
Mean [range]	75 [36–92]	79.4 [58–93]	84.9 [74–95]	77.6 [53–89]	80.5 [58–93]	79.3 [36–95]
*Gender, n (%)*						
Men	14 (66.7)	12 (63.2)	14 (82.4)	27 (79.4)	21 (58.3)	88 (69.3)
*Corrected Charlson score*						
Median [Q1,Q3]	2 [2–4]	3 [1–4.5]	3 [2–4]	3 [2–4]	2.5 [1.7–4]	3 [2–4]
*Immunosuppression, n (%)*						
Yes	4 (19.0)	8 (42.1)	2 (11.8)	12 (35.3)	9 (25.0)	35 (27.6)
*Details immunosuppression, n (%)*						
Renal transplant	1 (25.0)	3 (75.0)	0 (0.0)	2 (20.0)	1 (11.1)	7 (24.1)
Liver transplant	0 (0.0)	0 (0.0)	0 (0.0)	0 (0.0)	1 (11.1)	1 (3.4)
Myelodysplastic syndrome	0 (0.0)	0 (0.0)	1 (50.0)	2 (20.0)	1 (11.1)	4 (13.8)
chronic lymphocytic leukemia	3 (75.0)	1 (25.0)	0 (0.0)	5 (50.0)	4 (44.4)	13 (44.8)
Myeloma	0 (0.0)	0 (0.0)	0 (0.0)	0 (0.0)	1 (11.1)	1 (3.4)
Lymphoma	0 (0.0)	0 (0.0)	1 (50.0)	0 (0.0)	1 (11.1)	2 (6.9)
HIV/AIDS	0 (0.0)	0 (0.0)	0 (0.0)	1 (10.0)	0 (0.0)	1 (3.4)
Immunosuppressive treatment	3 (75.0)	8 (100.0)	1 (50.0)	8 (80.0)	6 (75.0)	26 (81.2)
*Other neoplastic history, n (%)*						
Yes	7 (33.3)	8 (42.1)	4 (23.5)	18 (52.9)	16 (44.4)	53 (41.7)
*Other antineoplastic therapy*						
Yes	2 (9.5)	2 (10.5)	1 (5.9)	6 (17.6)	2 (5.6)	13 (10.2)
*Topography, n (%)*						
Head and neck	9 (42.9)	12 (63.1)	14 (82.4)	23 (67.6)	27 (75.0)	85 (66.9)
Limbs	5 (23.8)	1 (5.3)	2 (11.7)	4 (11.8)	5 (13.9)	17 (13.4)
Multiples topographies	4 (19.0)	3 (15.8)	1 (5.8)	4 (11.8)	1 (2.8)	13 (10.2)
Chronic wound	2 (9.5)	2 (10.5)	0 (0.0)	2 (5.9)	2 (5.6)	8 (6.3)
Trunk	1 (4.8)	1 (5.3)	0 (0.0)	1 (2.9)	1 (2.8	4 (3.1)
*Initial R1 cSCC excision, n (%)*						
Yes	4 (36.4)	4 (28.6)	2 (22.2)	14 (50.0)	12 (37.5)	36 (33.5)
NA	10	5	8	6	5	34
*Pathological perineural invasion, n (%)*						
Yes	3 (30.0)	5 (35.7)	1 (10)	13 (48.1)	12 (37.5)	34 (36.6)
NA	11	5	7	7	4	34
*Management before systemic treatment, n (%)*						
No treatment	2 (9.5)	3 (15.8)	6 (35.3)	5 (14.7)	2 (5.6)	18 (14.2)
Surgery	12 (57.1)	7 (36.8)	5 (29.4)	12 (35.3)	23 (63.8)	59 (46.5)
Surgery + radiotherapy	6 (28.6)	5 (26.3)	2 (11.8)	12 (35.3)	9 (25)	34 (26.8)
Radiotherapy	1 (4.8)	4 (21.0)	4 (23.5)	5 (14.7)	2 (5.6)	16 (12.6)

*acSCC* advanced squamous cell carcinomas; *AIDS* acquired immune deficiency syndrome; *cSCC* squamous cell carcinomas; *HIV* human immunodeficiency virus; *NA* not available; *range* minimum and maximum value

**Fig. 2:**
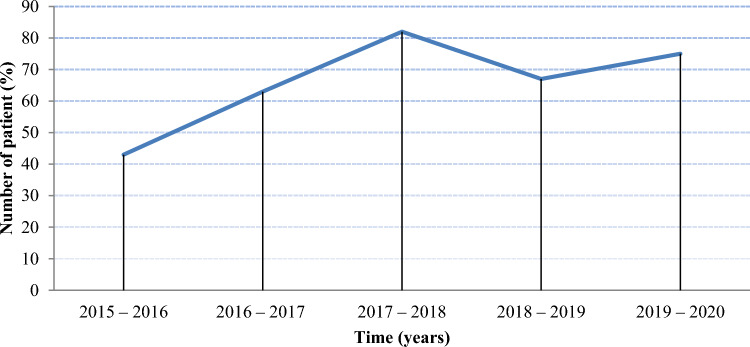
Trends in the numbers of head and neck cases per year during the 5-year study period

Most primary tumors were located on the head and neck (66.9%) and the incidence rate ratio of head and neck topography increased 33.1% each year (95% CI = [1.139; 1.565], *p* < 0.005, Fig. 2). Thirteen patients had cSCC at multiple sites (10). Eight patients (three women, five men) had acSCC on a chronic wound (6%), including lower limb ulcers (*n* = 4), breast cancer surgery and radiotherapy scars (*n* = 2), and chronic eschar (*n* = 2). Of the 127 patients, 34 had no available histological data on the initial cSCC, either because they had no previous surgery (*n* = 25) or the histological report was not available (*n* = 9). Of the remaining 93 patients, 34 had perineural invasion and 36 had R1 with positive excision margins.

### Initial cSCC management

Multidisciplinary meetings for initial cSCC management occurred in 70/127 cases, and the proposed management was applied in 92.8% of these cases. For initial care, 59 patients had surgery, 16 had radiotherapy exclusively, 34 had surgery plus radiotherapy, and 18 had no previous therapy (Table 1). Of the 48 patients with R1 excision or histological perineural invasion, only 31 were discussed in a multidisciplinary meeting (64.5%).

### Clinical characteristics of diseases at the start of systemic therapy

Table 2 summarizes the characteristics of the patients and their diseases. At the start of systemic therapy, the mean patient age was 81.6 (range 48–95) years, increasing by an average of 0.7 year annually, although this increase was not significant (*p* = 0.19). Of the 127 patients, 38 had local acSCC (29.9%), 63 had locoregional invasion (49.6%), and 26 had distant metastasis (20.5%). Distant metastases were in the lungs (*n* = 13), bones (*n* = 5), liver (*n* = 3), adrenal glands (*n* = 2), muscles (*n* = 2), digestive tract or peritoneal area (*n* = 2), heart (*n* = 1), and central nervous system (*n* = 1). There was no significant difference in first-line treatment over time (*p* = 0.69); 57 patients (44.9%) received platinum agents plus cetuximab and 56 patients (44.1%) received cetuximab alone. The mean time from cSCC diagnosis to the first perfusion was 27.1 (range, 0–240) months. Patients received a median of 2 (IQR 1–2) courses of systemic therapy; 32% of patients received immunotherapy at some point during their management. Therapeutic modification was due to disease progression (60.9%), disease stability (18.8%), poor treatment tolerance (15.6%), and altered general condition (4.7%).

**Table 2:** Medical management of localy advanced and metastatic cSCC

	2015–2016 (21)	2016–2017 (19)	2017–2018 (17)	2018–2019 (34)	2019–2020 (36)	TOTAL (127)
*Age at the beginning of systemic therapy, years*						
Mean [range]	78.1 [48–93]	81.3 [58–94]	86.3 [75–95]	80.4 [54–93]	82.7 [63–94]	81.6 [48–95]
*Stage of disease, n (%)*						
Locally advanced	4 (19)	5 (26.3)	6 (35.3)	12 (35.3)	11 (30.6)	38 (29.9)
Lymph node or parotid metastasis	14 (66.7)	9 (47.4)	6 (35.3)	16 (47)	18 (50.0)	63 (49.6)
Distant metastases	3 (14.3)	5 (26.3)	5 (29.4)	6 (17.7)	7 (19.4)	26 (20.5)
*Time from diagnosis to initiation systemic therapy (months)*						
Mean [range]	36.8 [2–177]	20.4 [1–114]	18.3 [0–117]	30.6 [0–223]	25.9 [0–241]	27.1 [0–241]
*Front-line treatment, n (%)*						
Platinum agent + Cetuximab	10 (47.6)	7 (36.8)	7 (41.2)	12 (35.3)	21 (58.3)	57 (44.9)
Cetuximab	9 (42.9)	9 (47.4)	9 (52.9)	16 (47.1)	13 (36.1)	56 (44.1)
Platinum agent + Cetuximab + 5FU	2 (9.5)	2 (10.5)	1 (5.9)	2 (5.9)	0 (0.0)	7 (5.5)
Cemiplimab	0 (0.0)	0 (0.0)	0 (0.0)	1 (2.9)	2 (5.6)	3 (2.4)
Pembrolizumab	0 (0.0)	0 (0.0)	0 (0.0)	2 (5.9)	0 (0.0)	2 (1.6)
Carboplatin + taxanes	0 (0.0)	1 (5.3)	0 (0.0)	1 (2.9)	0 (0.0)	2 (1.6)
*Anti-PD1 immunotherapy at any time, n (%)*						
Yes	1 (4.7)	0	2 (11.8)	15 (44.1)	23 (63.9)	41 (32.3)
*Total treatment duration (months)*						
Mean [range]	9.95 [0–40]	6.12 [4–42]	5.12 [0–28]	9.56 (0–29]	5.86 [0–17]	7.48 [0–42]
*Follow-up (months)*						
Median [IQR]	11 [8–26]	9 [3.5–14.5]	4 [3–23]	16.5 [5–24.3]	8 [5–11]	9 [4–17]

*IQR* interquartile range; *NA* not available; *PR* partial response; *range* minimum and maximum value

### Safety

Twenty-seven patients experienced an AE (21.4%), including 17 patients with AE grade ≥ 3. Of those with AE grade ≥ 3, one patient died due to the AE (immuno-induced myositis and respiratory distress) and 12 patients were admitted to hospital; six had been treated with cetuximab (four anaphylactic reactions, one giant urticaria, and one cardiac decompensation), one with carboplatin and cetuximab (hospitalized for a transfusion for symptomatic anemia), and five with cemiplimab (one each with colitis, sclerosing cholangitis, drug reaction with eosinophilia and systemic symptoms, renal failure, and myositis).

### Response rate, survival, and follow-up

The median follow-up was 9 (IQR 4–17) months. Patients received treatment for a mean of 7.48 (range 0–42) months; 45 patients (35.7%) were still being treated at the last evaluation (Table 2).

**Fig. 3:**
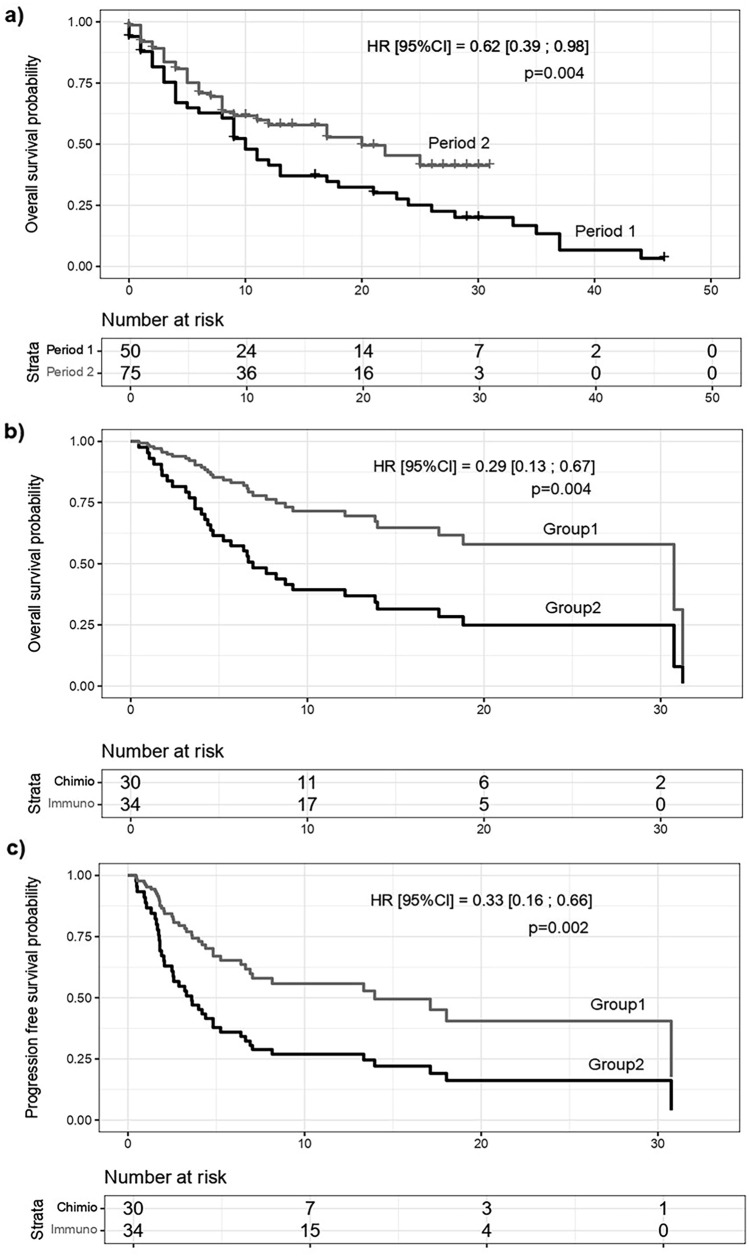
Survival analysis. **a** Overall survival (OS) in periods 1 and 2. **b** Cox model comparing OS in patients treated with immunotherapy (group 1) with those treated with other therapies (group 2) as second-line treatment. **c** Cox model comparing progression-free survival (PFS) in groups 1 and 2

The median OS was significantly higher in period 2 than in period 1 (20 vs. 10 months; hazard ratio [HR] [95% CI] = 0.62 [0.39; 0.98], *p* = 0.04; Fig. 3a). The median PFS was similar in the two periods (108 vs. 127 days, respectively; HR [95% CI] = 0.74 [0.50; 1.10], *p* = 0.13). The multivariate Cox model analysis showed a 61% reduction in the risk of death in patients who received immunotherapy (HR [95% CI] = 0.39 [0.22; 0.70], *p* = 0.002) at any time. Immunosuppressed patients had a 1.77-fold increase in the risk of death (HR [95% CI] = 1.77 [1.00; 3.13], *p* = 0.05) and a 1.69-fold increase in the risk of recurrence or progression (HR [95% CI] = 1.69 [1.03; 2.78], *p* = 0.04). The risk of progression increased with age (HR [95% CI] = 1.37 [1.07; 1.75], *p* = 0.01).

Among patients who received second-line treatment (*n* = 64), we compared those who received immunotherapy (group 1, *n* = 38) with those who received other therapies (group 2, *n* = 26) (Fig. 3b, c). Eight immunocompromised patients received immunotherapy. Subgroup analyses showed that PD-1 immunotherapy reduced mortality and progression by 71% (HR [95% CI] = 0.29 [0.13; 0.67] *p* = 0.004) and 67% (HR [95% CI] = 0.33 [0.16; 0.66] *p* = 0.002), respectively (Fig. 3b, c). The risk of progression increased 1.53-fold when the patients were older (HR [95% CI] = 1.53 [1.04; 2.24], *p* = 0.03) and 4.5-fold when immunosuppressed (HR [95% CI] = 4.50 [1.70; 11.88], *p* = 0.002). The sensitivity analysis, which excluded organ transplant patients, revealed the same results, with a 71% reduction in the risk of death and 66% reduction in the risk of relapse with second-line immunotherapy compared with other treatments (HR [95% CI] = 0.29 [0.13–0.67], *p* = 0.004 and 0.34 [0.16–0.69], *p* = 0.003).

## Discussion

Over the 5-year period, the majority of the patients with acSCC were very frail, elderly, predominantly males, with comorbidities, and more than 25% were immunocompromised. We observed an increase in patient numbers over time, with a mean increase of nearly 20% per year. Patients and their cSCC characteristics were similar over the 5-year period, except that the proportion with a head and neck location tended to increase with time.

Our study showed a clear improvement in OS between the first and second periods, with a twofold improvement in survival. Furthermore, mortality was clearly reduced in patients who received anti-PD-1 therapy compared to those who did not. Immunotherapy has revolutionized the management of some cancers, including acSCC, and cemiplimab was approved after nonrandomized trials (Migden et al. 2018). The pivotal phase II study included 59 patients; the overall response rate (ORR) was 47% [95% CI (34–61)] and the rate of durable disease control (DDC) was 61%. Based on these results, cemiplimab was approved by the FDA in September 2018 and by the EMA in July 2019 for acSCC ineligible for surgery or radiotherapy. In France, there was an early access program to cemiplimab from August 2018 to January 2021 to treat advanced or metastatic cSCC as second-line treatment or as first-line if ineligible for platinum-based chemotherapy. Pembrolizumab was also approved after nonrandomized trials (Maubec et al. 2020; Grob et al. 2020). However, in real-life studies, cemiplimab efficacy results were similar to the results of the phase II trials (Hober et al. 2021; Samaran et al. 2023). A French and Italian multicenter cohort study including respectively 240 (mean age 77 years) and 131 (median age of 79 year) patients showed an ORR of 50.4% and 42.7% (Baggi et al. 2021; Hober et al. 2021). In both studies, head and neck location were significantly associated with a better response. Severe treatment-related AE occurred in arrow 9% of both studies, including a total of 3 deaths. European guidelines and an overview proposed more recently, recommend cemiplimab as first-line treatment in patients with acSCC who are not candidates for curative surgery or radiation (Stratigos et al. 2020; Rubatto et al. 2023). It is important to remember that patients and their care remain complex and heterogeneous, and that it is necessary to define standardized care for this fragile, high-needs patient population (Mannino et al. 2023).

These highly promising results in the management of acSCC are associated with the emergence of immune-related AE that can affect many organs. In most cases, these immune-related AE can be managed (Gambichler et al. 2022). In our cohort, one patient dead immunotherapy-related death (myositis). Our acSCC patients are fragile, and it is important to assess the benefit-risk balance of immunotherapy, which can have severe, sometimes irreversible side effects. In our study, immunosuppression, which was present in 27.6% of patients, was the second most prominent factor modifying OS, with a 1.77-fold increase in the risk of death (HR [95% CI] = 1.77 [1.00; 3.13], *p* = 0.05). These patients mainly had hematological neoplasms (51.4%) or organ transplantation (22.8%), usually kidney transplantation. We observed that 37% of the patients with hematological diseases responded to immunotherapy despite immunosuppression, confirming that anti-PD-1 is a good therapeutic option in these patients. Conversely, anti-PD-1 antibodies were not used in transplant patients in this study, because the risk of acute or chronic rejection may lead to death (Tsung et al. 2021). The use of immunotherapy in transplanted patients begin to be reported. A systematic review of the literature, showed that the ORR was 34.5% for all types of cancer, and was significantly better in acSCC with an ORR of 68.2% (15/22). Transplant rejection occurred in 41.2% of cases, graft failure in 23.5% and immune-related AE in 18.5% (Portuguese et al. 2022). These data suggested that adapting anti-rejection therapies and prophylactic corticosteroid may reduce the risk of rejection (Rubatto et al. 2021; Tsung et al. 2021). Short-term efficacy looks promising, but prospective studies with more in-depth follow-up and a standardized protocol are needed.

cSCC and acSCC are major public health concerns due to the large number of patients and social and psychological consequences of acSCC, which include visible skin tumors on the head and neck. Actions that may reduce invasive SCC development (reduced sun exposure, early diagnosis) and better initial management of cSCC cases may decrease the incidence of acSCC. In our cohort, only 55.1% of patient records were discussed in a multidisciplinary meeting at the initial stage and only 26.8% of the 61 patients who had an indication for radiotherapy had access. Furthermore, a retrospective study of German and Austrian populations over a 1-year period analyzed data from 190 patients with cSCC; the results showed that 76 patients had locally advanced cSCC (40%) and 114 had metastatic cSCC (60%) (Hillen et al. 2018). Once diagnosed, most patients (59%) did not receive any therapy and only 32 patients (16.8%) received systemic antitumor therapies. The knowledge of physicians (surgeons, general practitioners, and dermatologists) who initially treat cSCC in these patients should be improved, and systematic discussion of such cases in multidisciplinary meetings could contribute to halting the increased incidence of acSCC.

The strength of this study lies in its unique nature, as epidemiological data on cSCC and acSCC are very scarce. Indeed, the latest national report with incidence and mortality estimates of cancer in metropolitan France covers the period from 1990 to 2018 (Deffossez et al. 2019), and provides no data on cSCC because it excludes NMSC. Although our study was based on a small patient cohort, the number of patients treated for carcinoma appears to have increased during the 5-year period. Advanced age and immunosuppression were associated with lower PFS. Overall survival was better in the most recent period and was significantly better in patients who received immunotherapy.

## Data availibility

No dataset were generated during this study.

## Abbreviations:

(a) cSCC: (Advanced) cutaneous squamous cell carcinoma
AE: Adverse event
CI: Confidence interval
CR: Complete response
DDC: Durable disease control
EGFR (i): Epidermal growth factor receptor (inhibitor)
EMA: European medicines agency
FDA: Food and drug administration
IQR: Interquartile range
NMSC: Non-melanoma skin cancers
ORR: Objective response rate
OS: Overall survival
PD-1: Programed cell-death protein-1
PFS: Progression-free survival
PR: Partial response
RECIST: Response evaluation criteria in solid tumors
